# Requirement of extracellular signal-regulated kinase/mitogen-activated protein kinase for long-term potentiation in adult mouse anterior cingulate cortex

**DOI:** 10.1186/1744-8069-3-36

**Published:** 2007-12-01

**Authors:** Hiroki Toyoda, Ming-Gao Zhao, Hui Xu, Long-Jun Wu, Ming Ren, Min Zhuo

**Affiliations:** 1 Department of Physiology, University of Toronto, 1 King's College Circle, Toronto, Ontario M5S 1A8, Canada

## Abstract

Long-term potentiation (LTP) in the anterior cingulate cortex (ACC) is believed to be critical for higher brain functions including emotion, learning, memory and chronic pain. N-methyl-D-aspartate (NMDA) receptor-dependent LTP is well studied and is thought to be important for learning and memory in mammalian brains. As the downstream target of NMDA receptors, the extracellular signal-regulated kinase (ERK) in the mitogen-activated protein kinase (MAPK) cascade has been extensively studied for its involvement in synaptic plasticity, learning and memory in hippocampus. By contrast, the role of ERK in cingulate LTP has not been investigated. In this study, we examined whether LTP in ACC requires the activation of ERK. We found that P42/P44 MAPK inhibitors, PD98059 and U0126, suppressed the induction of cingulate LTP that was induced by presynaptic stimulation with postsynaptic depolarization (the pairing protocol). We also showed that cingulate LTP induced by two other different protocols was also blocked by PD98059. Moreover, we found that these two inhibitors had no effect on the maintenance of cingulate LTP. Inhibitors of c-Jun N-terminal kinase (JNK) and p38, other members of MAPK family, SP600125 and SB203850, suppressed the induction of cingulate LTP generated by the pairing protocol. Thus, our study suggests that the MAPK signaling pathway is involved in the induction of cingulate LTP and plays a critical role in physiological conditions.

## Introduction

The prefrontal cortex, including the anterior cingulate cortex (ACC) is believed to play important roles in emotion, learning, memory and persistent pain in the adult brain [1-7]. Long-term potentiation (LTP), known to be involved in learning and memory, is a key synaptic mechanism for cortical synaptic plasticity [8]. Recent studies demonstrate that LTP can be induced in the cingulate slices [3,9,10]. However, several recent studies showed that molecular signaling pathways involved in the synaptic potentiation in the ACC differ from those in the hippocampus. For example, both N-methyl-D-aspartate (NMDA) receptor subunit 2A and 2B (NR2A and NR2B) contribute to cingulate LTP [3], while NR2A is preferentially contributing to hippocampal LTP [11,12]. For calcium-related signaling messengers, calcium-calmodulin (CaM) dependent adenylyl cyclase (AC) type 1 is critical for synaptic LTP in the ACC [9], while AC1 deletion alone did not affect hippocampal LTP [13]. On the other hand, the downstream targets of calcium-stimulated cAMP-dependent signaling pathways underlying LTP in the ACC synapses have been far less investigated compared to hippocampal synapses.

As the downstream target of cAMP signaling pathways, mitogen-activated protein kinase (MAPK) is well characterized in the hippocampus [14,15]. The MAPK is a family of serine/threonine protein kinases that transduce extracellular signals from cell surface receptors to the cell nucleus [16,17]. The MAPK cascade includes extracellular signal-regulated (ERK), p38, c-Jun N-terminal kinase (JNK), and ERK5 [17]. The activation of ERK is coupled to stimulation of cell surface receptors via several different upstream signaling pathways, and plays critical roles in the regulation of gene expression and cell proliferation [18]. In neurons, the ERK signaling pathway is activated by synaptic activity such as membrane depolarization, calcium influx and neurotrophins [19-21]. Furthermore, the ERK signaling pathway might regulate synaptic targets to control important functions such as synaptic plasticity, learning and memory in the adult brain [15,22,23]. However, the role of ERK signaling pathway in the cingulate synaptic plasticity has not been investigated.

In the present study, we performed whole-cell patch-clamp recordings from cingulate neurons of adult mice and investigated the role of MAPK in the cingulate synaptic potentiation. Here, we show that LTP induced by three different induction protocols were completely blocked by the MAPK/ERK kinase (MEK) inhibitor applied postsynaptically. Furthermore, we found that the MEK inhibitors did not affect the maintenance of cingulate LTP. Inhibitors of c-Jun N-terminal kinase (JNK) and p38 also suppressed the induction of cingulate LTP generated by the pairing protocol. These results suggest that the activation of MAPK including ERK, JNK and p38, is critical for the induction of LTP in the ACC.

## Results

### Postsynaptic injection of MAPK inhibitors blocks the cingulate LTP

**Figure 1:**
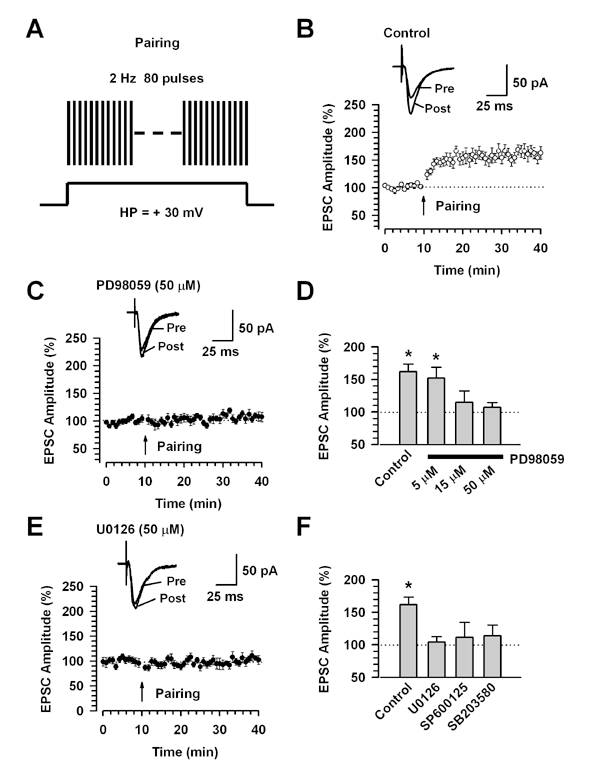
**Postsynaptic inhibition of PD98059 prevents the induction of cingulate LTP**. A: A scheme illustrating the LTP induction protocol consisting of pairing 80 presynaptic pulses at 2 Hz with postsynaptic depolarization (holding at + 30 mV). B: LTP is induced by the pairing protocol in cingulate pyramidal neurons (n = 13). C: The MEK inhibitor, PD98059 (50 μM, n = 10) in the intracellular solution completely blocks LTP induction. Traces show averages of six EPSCs at baseline response (pre) and 30 min (post) after the pairing protocol (arrow). The dashed line indicates the mean basal synaptic response. D: Summary of the effects of PD98059 at different concentrations on LTP induced by the pairing protocol (PD98059; 5 μM, n = 8; 15 μM, n = 6, 50 μM, n = 10). * *P *< 0.05 compared to baseline response. E: The MEK inhibitor U0126 (50 μM) in the intracellular solution completely blocks LTP induction. Traces show averages of six EPSCs at baseline response (pre) and 30 min (post) after the pairing protocol (arrow). The dashed line indicates the mean basal synaptic response. F: Summary of the effects of MAPK inhibitors on LTP induced by the pairing protocol (U0126; n = 7, SP600125; n = 6, SB203850; n = 6). * *P *< 0.05 compared to baseline response.

We performed conventional whole-cell patch-clamp recordings from visually identified pyramidal neurons in the layer II/III of cingulate slices. Fast EPSCs were obtained by delivering focal electrical stimulation to the layer V. First, we identified pyramidal neurons based on the pyramidal shape of their somata by loading Lucifer yellow into the intracellular solution [3]. We also confirmed that the recordings were performed from cortical pyramidal cells by injecting depolarizing currents into the neuron. Injection of depolarizing currents into neurons induced repetitive action potentials with significant firing frequency adaptation [3]. Next, we carried out experiments to determine if the pairing of synaptic activity with postsynaptic depolarization (or called the pairing protocol) may induce long-term potentiation (LTP) of synaptic responses in the ACC. We induced LTP by pairing 80 presynaptic pulses at 2 Hz with postsynaptic depolarization (holding at + 30 mV) [3,24]. LTP was induced with the pairing protocol within 12 minutes after establishing the whole-cell configuration to avoid washout of intracellular contents that are critical for the establishment of synaptic plasticity [3]. Indeed, the pairing protocol produced a significant, long-lasting potentiation of synaptic responses (161.9 ± 11.7%, n = 13, *P *< 0.05 compared with baseline response, Fig. 1B, D).

In our previous study, we have shown that the expression of LTP in the ACC depends on a postsynaptic mechanism [3]. Therefore, we examined the effects of MAPK inhibitors on cingulate LTP by postsynaptic injection. We tested whether LTP induced by the pairing protocol is prevented by postsynaptic application of a MAPK inhibitor, PD98059 [25] (Fig. 1C, D). Postsynaptic injection of PD98059 (5 μM), in the intracellular solution had no effect on cingulate LTP induced by the pairing protocol (5 μM; 152.3 ± 16.8%, n = 8, *P *< 0.05 compared with baseline response, Fig. 1D). However, PD98059 at higher concentrations (15 and 50 μM) completely blocked the induction of cingulate LTP (15 μM; 114.8 ± 17.4%, n = 6, *P *> 0.05 compared with baseline response, 50 μM: 107.3 ± 7.4%, n = 10, *P *> 0.05 compared with baseline response, Fig. 1B, D). It has been reported that an alteration in AMPA receptor channel kinetics could underlie the expression of LTP [26]. Then, we analyzed the rise and decay times before and after the induction of LTP to examine whether LTP induced by the pairing protocol involves a change in the kinetics of the EPSCs. The rise and decay times of EPSCs showed no difference before and after the application of the pairing protocol (data not shown). Those of EPSCs were also not affected by the intracellular perfusion of PD98059 (50 μM) (data not shown).

We also used another MEK inhibitor U0126 (50 μM) in the intracellular solution [27]. Postsynaptic application of U0126 completely blocked the induction of LTP generated by the pairing protocol (U0126; 104.4 ± 8.1%, n = 7, *P *> 0.05 compared with baseline response, Fig. 1E, F). Then we tested the effects of JNK or p38 inhibitor on the induction of cingulate LTP, because the MAPK signaling pathways include extracellular signal-regulated (ERK), c-Jun N-terminal kinase (JNK), p38 and ERK5 [17]. Similar to MEK inhibitors, a JNK inhibitor, SP600125 (10 μM) [28] or a p38 inhibitor, SB203580 (10 μM) [29] significantly suppressed the induction of cingulate LTP (SP600125: 111.3 ± 17.1%, n = 6, *P *> 0.05 compared with baseline response; SB203580: 114.0 ± 16.6%, n = 6, *P *> 0.05 compared with baseline response, Fig. 1F). Since PD98059 and U0126 have been reported to also inhibit MEK5, the upstream regulator of ERK5 [30], these results suggest that the activation of all MAPK signaling pathways is required for the induction of cingulate LTP. However, we can not completely rule out possible non-selective effects of pharmacological inhibitors.

### Inhibition is independent of the induction protocols

**Figure 2:**
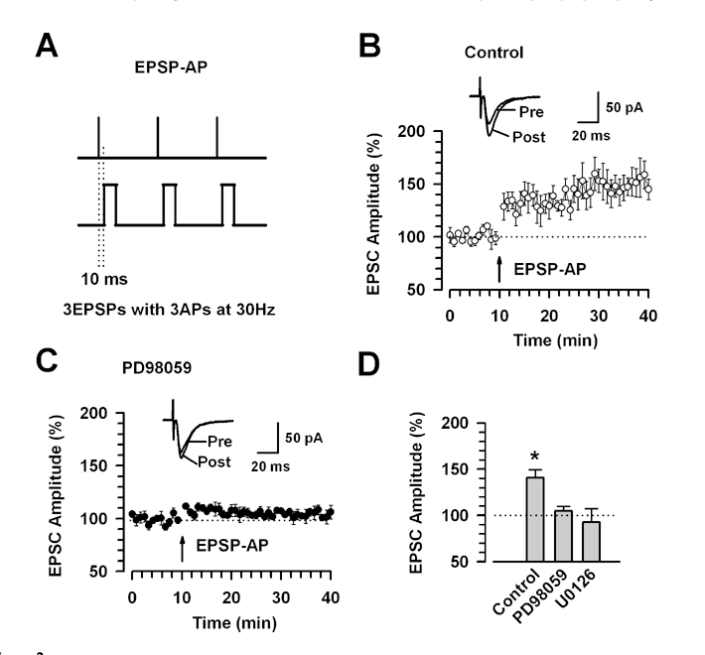
**LTP induced by EPSP-AP protocol is blocked by PD98059**. A: A scheme illustrating the LTP induction protocol consisting of 3 EPSPs and 3 APs (10 ms ahead) at 30 Hz, which are repeated 15 times with the interval of 5 s. B: LTP is induced by the EPSP-AP protocol. This protocol induces significant LTP in adult ACC neurons (n = 8). C: LTP is blocked by addition of PD98059 in the intracellular solution. Traces show averages of six EPSCs at baseline response (pre) and 30 min (post) after the EPSP-AP protocol (arrow). The dashed line indicates the mean basal synaptic response. D: Summary of the effects of PD98059 (50 μM, n = 7) and U0126 (50 μM, n = 6) on LTP induced by the EPSP-AP protocol. * *P *< 0.05 compared to baseline response.

To test whether the activation of ERK may depend on a specific LTP induction paradigm, we decided to test the role of PD98059 in cingulate LTP using two other different induction protocols. First, we tested a protocol of coincidence of postsynaptic action potentials (APs) and unitary EPSPs (or called EPSP-AP) to induce LTP [31,32] (Fig. 2A). This protocol is useful to test synaptic modifications, since precise spike timing may be used in the information processing in the neocortex [33]. Coincidence between EPSPs and backpropagating APs leads to the induction of LTP or LTD, depending on the timing of EPSPs and APs. Repetitive postsynaptic spiking within a time window of 10 ms after presynaptic activation resulted in LTP [31]. This protocol induced a significant, long-lasting potentiation of synaptic responses (140.6 ± 9.0%, n = 8, *P *< 0.05 compared with baseline response; Fig. 2B, D). The potentiation was completely blocked by 50 μM PD98059 or 50 μM U0126 in the intracellular solution (PD98059: 104.8 ± 5.1%, n = 7, *P *> 0.05 compared with baseline response; U0126: 92.6 ± 9.6%, n = 6, *P *> 0.05 compared with baseline response) (Fig. 2C, D).

**Figure 3:**
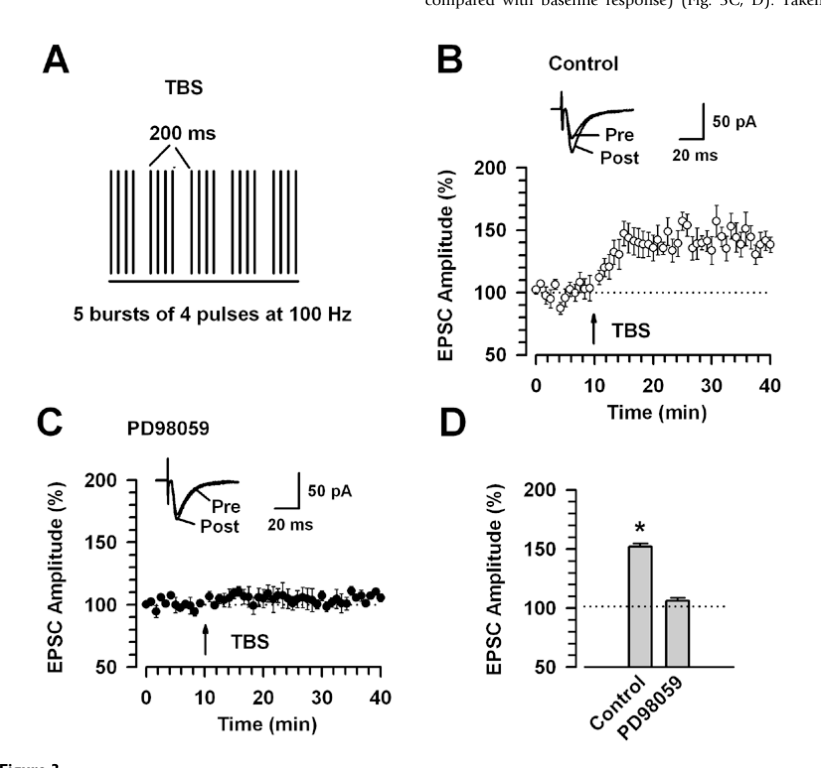
**LTP induced by TBS protocol is blocked by PD98059**. A: A scheme illustrating the LTP induction protocol consisting of 5 trains of burst with 4 pulses at 100 Hz, 200 ms interval, which are repeated 4 times with the interval of 10 s. B: LTP is induced by the TBS protocol. The TBS protocol induced significant LTP in the ACC of adult mice (n = 8). C: LTP is blocked by addition of PD98059 (50 μM) in the intracellular solution. Traces show averages of six EPSCs at baseline response (pre) and 30 min (post) after the TBS protocol (arrow). The dashed line indicates the mean basal synaptic response. D: Summary of the effects of PD98059 (50 μM, n = 6) on LTP induced by the TBS protocol. * *P *< 0.05 compared to baseline.

Next, we induced LTP using theta-burst stimulation (TBS) [3,34] (Fig. 3A). This paradigm is thought to be physiological, since the synchronized firing patterns at similar frequencies are observed during learning in the hippocampus [35]. We found that TBS induced significant LTP in the cingulate neurons (151.8 ± 12.7%; n = 8, *P *< 0.05 compared with baseline response) (Fig. 3B). The induction of LTP was also blocked by 50 μM PD98059 in the intracellular solution (106.1 ± 2.6%; n = 6, *P *> 0.05 compared with baseline response) (Fig. 3C, D). Taken together, these results indicate that the activation of ERK in LTP induction is not dependent on specific induction paradigms.

### LTP can be induced in the absence of picrotoxin

**Figure 4:**
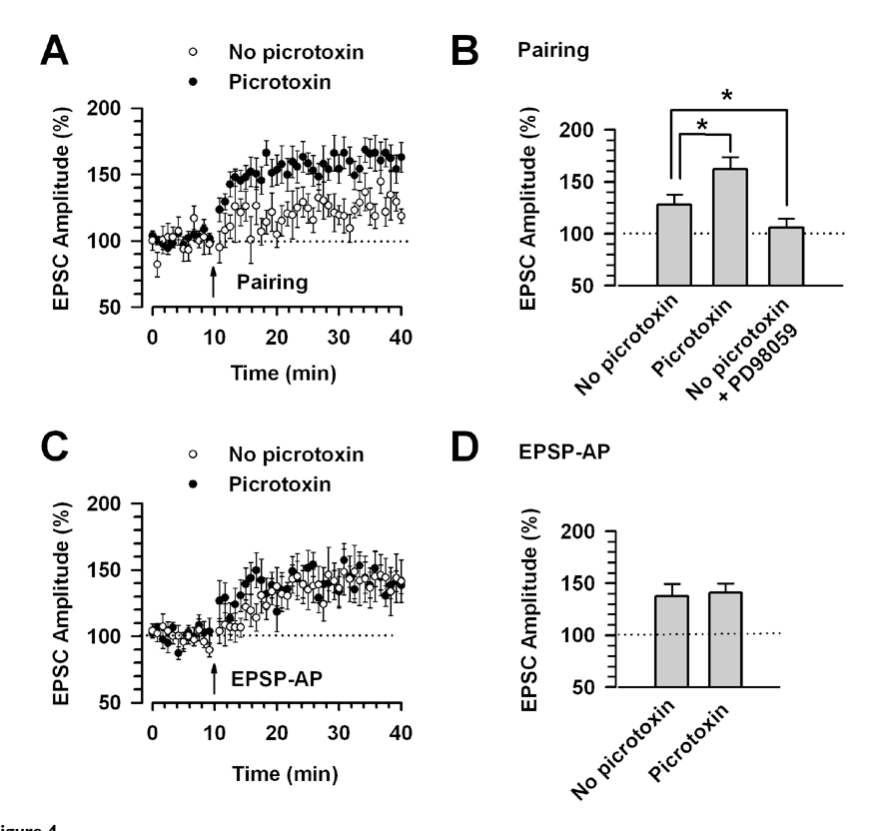
**LTP induced by pairing and EPSP-AP protocols in the absence and presence of picrotoxin**. A, B: In the absence of picrotoxin (No picrotoxin), LTP induced by the pairing protocol (n = 8) is significantly reduced compared to that in the presence of picrotoxin (Picrotoxin; n = 13, see Fig. 1B, D). PD98059 (50 μM, n = 6) blocks the pairing-induced LTP in the absence of picrotoxin (No picrotoxin + PD98059). * *P *< 0.05 compared with EPSC amplitude (%) in the absence of picrotoxin (No picrotoxin). C, D: LTP induced by the EPSP-AP protocol shows no difference between in the absence (No picrotoxin, n = 9) and presence (Picrotoxin, n = 8, see Fig. 2B, D) of picrotoxin.

Previous studies indicate that LTP of glutamatergic synapses in the lateral amygdala is controlled by GABA_A _receptor-mediated inhibition (Shumyatsky et al, 2002; Bissiere et al, 2003). Thus, we examined whether the pairing or EPSP-AP protocol induces LTP in ACC synapses in the absence of picrotoxin. We found that LTP in the ACC was induced by the pairing protocol, even in the absence of picrotoxin (127.8 ± 10.0%, n = 8, *P *< 0.05 compared with baseline response, Fig. 4A, B). However, LTP induced by the pairing protocol in the absence of picrotoxin was significantly smaller than that in the presence of picrotoxin (see Fig. 1B, D) (*P *< 0.05, Fig. 4B). Postsynaptic application of PD98059 (50 μM) in the absence of picrotoxin blocked the pairing-induced LTP (106.1 ± 8.3%, n = 6, *P *> 0.05 compared with baseline response, Fig. 4B).

Next, we examined the effect of picrotoxin on LTP induced by the EPSP-AP protocol. Our results showed that LTP induced by the EPSP-AP protocol showed no difference between in the absence (137.6 ± 11.5%, n = 9, *P *< 0.05 compared with baseline response) and presence (140.6 ± 9.0%, see Fig. 2B, D) of picrotoxin (Fig. 4C, D). These results indicate that different LTP induction protocols would cause different inhibitory actions in the ACC synapses.

### ERK inhibitors do not affect AMPA receptor-mediated baseline EPSCs

In the following series of experiments, we used ERK inhibitors by bath application to test whether these drugs affect basal synaptic transmission, because it is reported that activation of presynaptic MAPK may enhance synaptic vesicle recycling and regulate short-time presynaptic plasticity in cultured hippocampal neurons [36].

**Figure 5:**
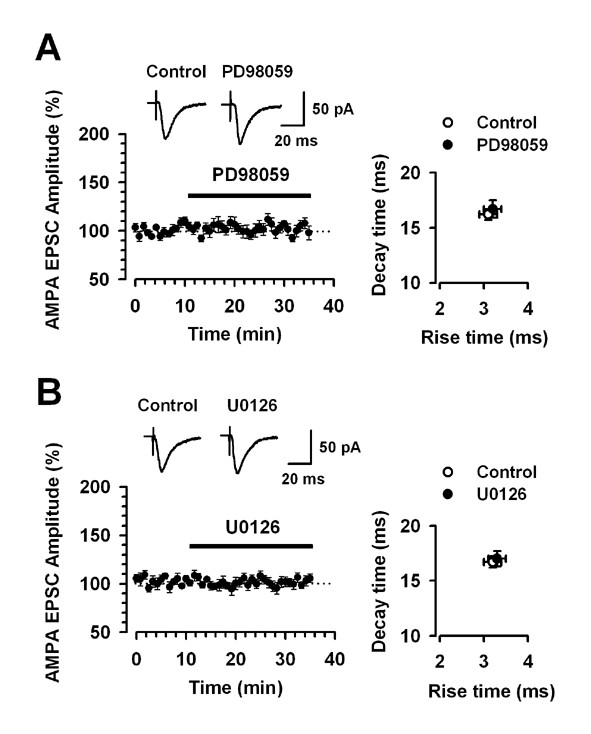
**MEK inhibitors do not affect the AMPA receptor-mediated EPSCs**. A, B: Bath application of PD98059 (50 μM, n = 10) or U0126 (50 μM, n = 6) does not affect the AMPA-receptor-mediated EPSCs. The insets show averages of six EPSCs before (Control; open) and after (PD98059 or U0126; solid) application of PD98059 or U0126. The dashed line indicates the mean basal synaptic response. Decay time versus rise time for AMPA receptor-mediated EPSCs before (Control; open) and after (PD98059 or U0126; solid) application of PD98059 or U0126.

First, we examined the effects of these inhibitors on AMPA receptor-mediated baseline EPSCs in cingulate slices. To record AMPA receptor-mediated EPSCs, we added AP-5 (50 μM) in the recording solution. Bath application of PD98059 or U0126 did not affect the AMPA receptor-mediated baseline EPSCs (PD98059 (100 μM): last 5 min of application, 101.5 ± 3.8 % of baseline response, n = 10, *P *> 0.05, Fig. 5A; U0126 (100 μM): last 5 min of application, 102.4 ± 4.6 % of baseline response, n = 6, *P *> 0.05, Fig. 5B). The rise and decay times of AMPA receptor-mediated EPSCs were not significantly altered during the recordings in the presence of PD98059 or U0126 (Fig. 5A, B).

### NMDA receptor-mediated baseline EPSCs

**Figure 6:**
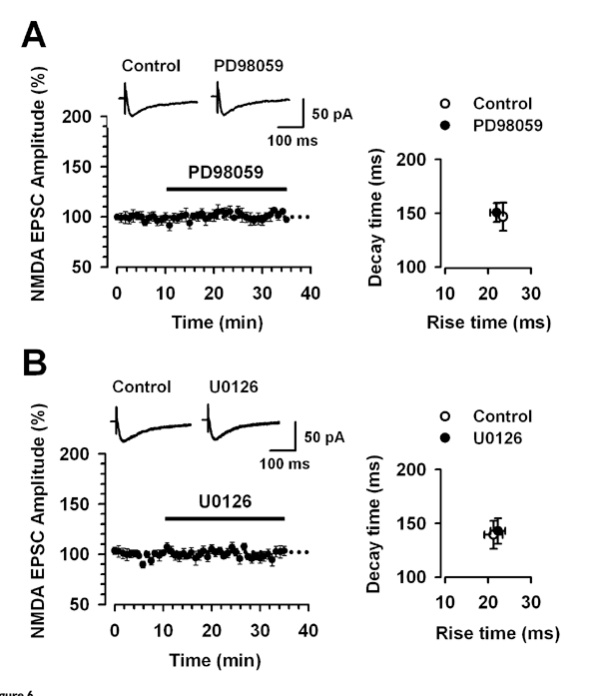
**MEK inhibitors do not affect the NMDA receptor-mediated EPSCs**. A, B: Bath application of PD98059 (100 μM, n = 7) or U0126 (100 μM, n = 7) does not affect NMDA receptor-mediated EPSCs. The insets show averages of six NMDA receptor-mediated EPSCs before (Control; open) and after (PD98059 or U0126; solid) application of PD98059 or U0126. Decay time versus the rise time for NMDA receptor-mediated EPSCs before (Control; open) and after (PD98059 or U0126; solid) application of PD98059 or U0126.

NMDA receptors are critical for the induction of cingulate LTP [3]. To test the possibility that MEK inhibitors affect the induction of LTP by inhibiting NMDA receptor-mediated currents, we examined the effects of the MEK inhibitors on synaptically induced NMDA receptor-mediated baseline EPSCs. We applied the MEK inhibitors in the extracellular solution after recording the baseline current responses. As we have shown in Fig. 6, NMDA receptor-mediated baseline EPSCs had not been changed by bath application of PD98059 or U0126 (PD98059 (100 μM): last 5 min of application, 102.1 ± 3.2 % of baseline response, n = 7, *P *> 0.05, Fig. 6A; U0126 (100 μM): last 5 min of application, 101.1 ± 5.2 % of baseline response, n = 7, p > 0.05, Fig. 6B). We further analyzed whether the MEK inhibitors affect the kinetics of NMDA receptor-mediated EPSCs. The rise and decay times of NMDA receptor-mediated EPSCs were not significantly altered during the recording in the presence of PD98059 or U0126 (Fig. 6A, B). These results suggest that PD98059 and U0126 do not inhibit LTP by simply inhibiting NMDA receptor function.

### Paired-pulse facilitation

**Figure 7:**
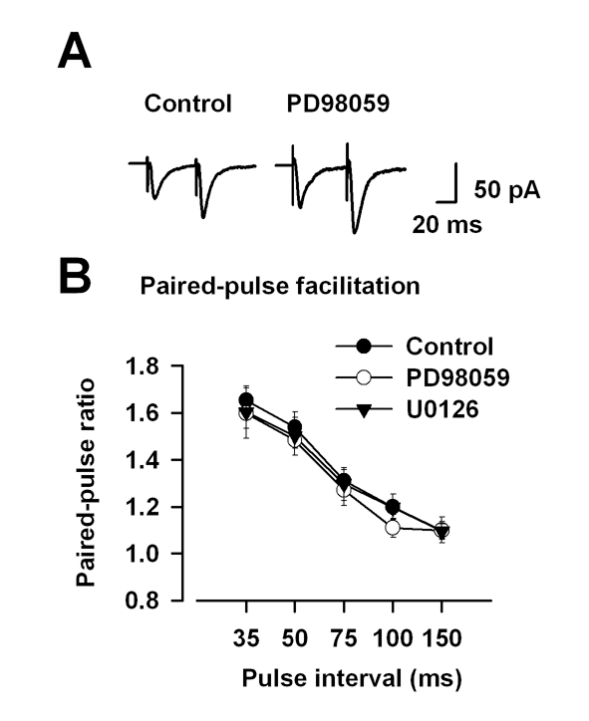
**MEK inhibitors do not affect the paired-pulse facilitation**. A: Sample traces show averages of six paired-pulse facilitation (PPF: the ratio of EPSC2/EPSC1) EPSCs at the 50 ms interval. B: PD98059 (100 μM, n = 7) or U0126 (100 μM, n = 6) in the extracellular solution has no effect on PPF.

We also examined the effect of PD98059 and U0126 on paired-pulse facilitation (PPF), a simple form of synaptic plasticity. Bath application of PD98059 (100 μM) or U0126 (100 μM), did not affect PPF at all time points (control, n = 13, PD98059, n = 7: U0126, n = 6) (Fig. 7). These results suggest that the MEK inhibitors had no effect on basal synaptic transmissions in ACC synapses.

**Figure 8:**
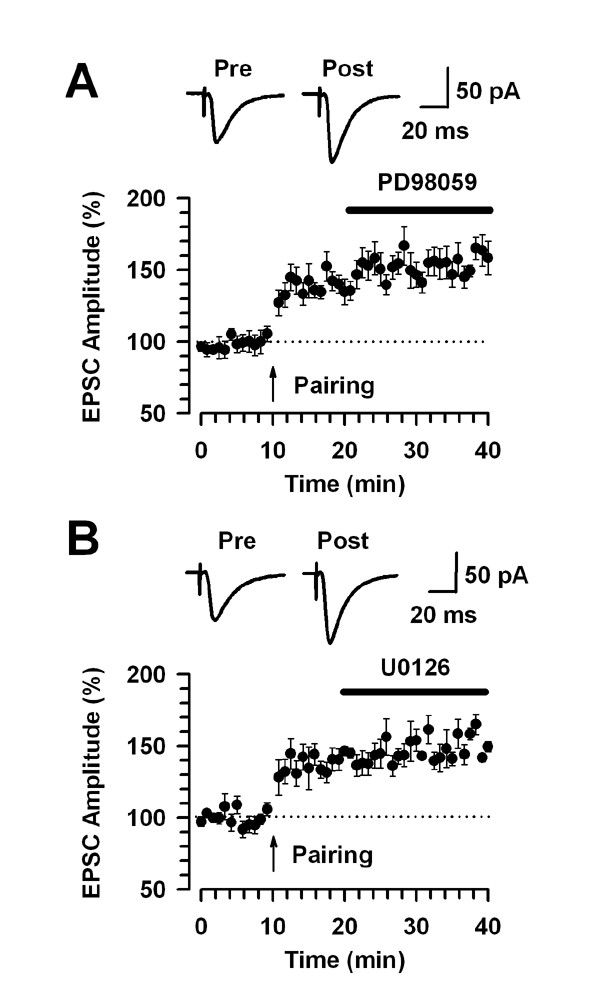
**MEK inhibitors do not affect the maintenance phase of LTP**. A, B: PD98059 (100 μM) or U0126 (100 μM) does not affect the maintenance of LTP (PD98059, n = 7: U0126, n = 5). The insets show averages of six EPSCs at baseline responses and 30 min after induction of the pairing protocol (arrow). The dashed line indicates the mean basal synaptic response.

The maintenance of LTPTo examine the effect of PD98059 and U0126 on the maintenance of LTP, PD98059 (100 μM) or U0126 (100 μM) was bath applied 10 min after the pairing protocol. In contrast to the application before the induction, we found no significant effect on the maintenance of LTP during the 20 min treatment with PD98059 or U0126 (PD98059, 156.5 ± 9.0 % at 30 min after the pairing protocol, 20 min after PD98059 treatment, n = 7, *P *< 0.05 compared with baseline response, Fig. 8A; U0126, 152.9 ± 5.7 % at 30 min after the pairing protocol, 20 min after U0126 treatment, n = 5, *P *< 0.05 compared with baseline response, Fig. 8B). These results suggest that the ERK inhibitors have no effect on the maintenance of cingulate LTP.

## Discussion

In this study, we demonstrated that ERK activation is required for the induction of LTP in the ACC and that the MEK inhibitors did not affect the maintenance phase of cingulate LTP. Furthermore, we showed that inhibitors of other members of MAPK family, such as JNK and p38, also blocked the induction of cingulate LTP generated by the pairing protocol. Thus, ERK/MAPK activation is essential for triggering long-term synaptic changes in the ACC, which plays critical roles in physiological and pathological conditions.

### The ERK activation in synaptic plasticity

The role of ERK in synaptic plasticity has been shown in several organisms including invertebrates and vertebrates. The ERK signaling pathway has been shown to be required for long-term facilitation of the sensory to motor synapse in the invertebrates, *Aplysia *[37]. On the other hand, the ERK signaling pathway has also been extensively studied in vertebrates, especially in mammalian brains (Table 1). The first evidence about the role of ERK activation in synaptic plasticity was shown in the CA1 region of the hippocampus [23], where NMDA-dependent LTP was blocked by a MEK inhibitor, PD98059. Thereafter, this phenomenon has been replicated by other studies [22,38,39]. The ERK activation is involved in NMDA receptor-independent LTP as well [40]. The involvement of ERK in synaptic plasticity has also been reported in a number of other brain areas. In the dentate gyrus, the ERK activity is required for multiple forms of synaptic plasticity including NMDA-dependent and NMDA-independent LTP [41], and such activity is necessary for *in vivo *LTP [25,42,43]. Furthermore, the ERK activation is essential for both memory consolidation of Pavlovian fear conditioning and synaptic plasticity in the lateral amygdala [44], which might be related to synthesis of new protein and mRNA [45]. In the cerebral cortex, the functional significance of the ERK signaling in synaptic plasticity has been well investigated. For instance, the ERK activation is involved in both synaptic plasticity and taste learning in the insular cortex [46]. Additionally, it has been reported that the blockade of ERK activation prevented LTP in the developing visual cortex and blocked the ocular dominance shift induced by monocular deprivation [47]. Recently, we have shown that the postsynaptic inhibition of the ERK pathway blocked LTP in superficial dorsal horn neurons [48], suggesting that the ERK activation in the superficial dorsal horn of the spinal cord can be pathophysiologically related to spinal sensitization and chronic pain after injury. Thus, the ERK signaling pathway is essential for many forms of synaptic plasticity. The ERK activation is also suggested to contribute to the formation of LTD as well as LTP in the prefrontal cortex, in which the ERK activation is required for LTD mediated by the coactivation of dopamine receptors and metabotropic glutamate receptors [49].

**Table 1:** The role of ERK in synaptic potentiation in the mammalian brains

**Brain region**	**Type of synaptic potentiation**	**References**
Hippocampus		
CA1	NMDA receptor-dependent LTP	[16], [23], [38], [39]
	NMDA receptor-independent LTP	[40]
		
Dentate gyrus	NMDA receptor-dependent LTP	[41]
	NMDA receptor-independent LTP	[41]
	*in vivo *LTP	[25, 42, 43]
Amygdala		[45], [64]
Visual cortex		[47]
Insular cortex	*in vivo *LTP	[46]
Cingulate cortex		The current work
Spinal cord		[48]

### The molecular mechanism of synaptic potentiation in the ACC

The molecular and cellular mechanisms of synaptic potentiation in the ACC are beginning to be elucidated by pharmacological and genetic studies. The neuronal activity triggered by LTP-inducing stimuli increases the release of glutamate in the cingulate synapses. The activation of NMDA receptors including NR2A and NR2B subunits and L-type voltage-gated calcium channels (L-VDCCs) induces an increase in postsynaptic calcium in dendritic spines [3,9]. Calcium influx via NMDA receptors and L-VDCCs plays a key role for triggering biological processes that lead to cingulate LTP. Postsynaptic calcium binds to calmodulin (CaM) and triggers various intracellular protein kinases and phosphatases [50]. CaM target proteins, such as Ca^2+^/CaM-dependent protein kinases (PKC, CaMKII and CaMKIV), CaM-activated ACs (AC1 and 8), and the CaM-activated phosphatase calcineurin, are known to be important for synaptic plasticity in the hippocampus [8,51,52]. Among them, activation of AC1 and CaMKIV have been reported to be essential for the induction of LTP in the ACC [4,9]. As the downstream target of AC1, cyclic-AMP(cAMP)-dependent protein kinase (PKA) has been well documented, which may activate MEK and ERK/MAPK via the activation of AC1. Activated ERK/MAPK likely has multiple targets including cAMP response element binding protein (CREB) that is required for long-term synaptic changes in neurons [4]. In the present study, JNK [28] or p38 inhibitor [29] blocked the induction of cingulate LTP generated by the pairing protocol, indicating that JNK and p38 would be involved in the induction of cingulate LTP. By contrast, in hippocampus, it has been reported that different MAPK cascades plays different roles for synaptic plasticity; Ras-Erk1/2 for LTP, Rap1-p38 for LTD, and Rap2-JNK for depotentiation [53].

LTP is typically divided into two phases such as early-phase and late-phase LTP (E-LTP and L-LTP, respectively). E-LTP depends on the activation of kinases and phosphatases, while L-LTP depends on the change of gene expression. Considering the importance of ERK in regulating gene expression, the ERK activation may be required for L-LTP. In previous reports, not only L-LTP but also E-LTP were inhibited by the MEK inhibitor, PD98059 [16,38]. Thus, the ERK signaling not only regulates the gene expression required for L-LTP, but also contributes to activation of several kinases required for E-LTP. In the present study, the maintenance of cingulate LTP was not affected by PD98059, suggesting that the ERK signaling cascade is not persistently activated during LTP in the ACC. This phenomenon is consistent with a previous report, in which PD98059 had no effect on the expression of LTP in the hippocampus [23]. The molecular mechanisms underlying the maintenance of LTP are not well understood. Calcium influx into the postsynaptic membrane is an essential process in the induction of LTP [54], and this enhanced calcium levels in the postsynaptic neurons activate several protein kinases including the CaMKII, which plays a pivotal role in the induction of LTP [38,55,56]. Autophosphorylation of the CaMKII leads the kinase into an autonomous mode of activity, and this molecular switch is believed to be important for experience-dependent synaptic plasticity, learning, and memory in the hippocampus [52,55]. Since protein kinase C (PKC) and CaMKII have been shown to be required for the induction, but not maintenance of hippocampal LTP [52,55], such autonomous activation of ERK might have contributed to the maintenance of cingulate LTP. Thus, the MEK inhibitors would have an inhibitory effect on the induction but not the maintenance of LTP. Taken together, the ERK activation is an important signaling cascade in triggering the synaptic potentiation in the ACC.

### Physiological and pathological significance

The prefrontal cortex, including the ACC, is thought to be important for higher brain functions in emotion, learning, memory and chronic pain [1-6,57]. Previous our studies using AC1 and AC8 double-knockout or NR2B overexpressed mice show that the AC1, AC8 and NR2B receptors in the ACC contribute to the behavioral allodynia [4,58]. Roles for the ACC in remote contextual fear memory [57] and spatial memory [1] have also been reported. By contrast, another line of evidence suggests that the ACC may play a critical role in the acquisition of fear memory [2,3]. Indeed, fear memory has been caused by direct stimulation of the ACC [2], and NR2B subunit in the ACC has been demonstrated to be involved in the induction of LTP and acquisition of contextual fear memory [3]. Although the ERK activation in the prefrontal cortex has been indicated to play a critical role in long-term memory storage [59], more studies are necessary to understand the roles of ERK in the formation of contextual fear memory and persist pain. Furthermore, it has been reported that the ERK activation in prefrontal cortex contributed to reward and aversion effects of drugs of abuse [60] and that ERK phosphorylation in the prefrontal cortex increased under chronic stress state [61]. Therefore, the ERK activation in the ACC is necessary for not only physiological but also pathological conditions. Understanding synaptic plasticity in the ACC will help us provide the new insight about cortical processing and memory formation under physiological and pathological conditions.

## Methods

### Animals and slice preparation

The Animal Care and Use Committee of University of Toronto approved the mouse protocols. C57BL/6 mice (6–8 weeks old) were anesthetized with halothane, and coronal brain slices (300 μM) containing the ACC were prepared using our previous methods [3,10,62]. Slices were transferred to a submerged recovery chamber with oxygenated (95% O_2 _and 5% CO_2_) artificial cerebrospinal fluid (ACSF) containing (in mM: 124 NaCl, 2.5 KCl, 2 CaCl_2_, 1 MgSO_4_, 25 NaHCO_3_, 1 NaH_2_PO_4_, 10 glucose) at room temperature for at least 1 h.

### Whole-cell patch-clamp recordings

Experiments were performed in a recording chamber on the stage of an Axioskop 2FS microscope with infrared DIC optics for visualization of whole-cell patch clamp recording. Neurons of the ACC in the layer II, III and V received afferent input from the thalamus [63]. In the present study, excitatory postsynaptic currents (EPSCs) were recorded from the layer II/III neurons with an Axon 200B amplifier (Molecular Devices, CA) and the stimulations were delivered by a bipolar tungsten stimulating electrode placed in the layer V of the ACC slices [3,10,62]. EPSCs were induced by repetitive stimulations at 0.02 Hz and neurons were voltage clamped at -70 mV. The recording pipettes (3–5 MΩ) were filled with solution containing (mM) 145 K-gluconate, 5 NaCl, 1 MgCl_2_, 0.2 EGTA, 10 HEPES, 2 Mg-ATP, and 0.1 Na_3_-GTP (adjusted to pH 7.2 with KOH). In the most of experiment, picrotoxin (100 μM) was present to block GABA_A _receptor-mediated inhibitory currents. In some experiment, LTP was induced in the absence of picrotoxin. Three kinds of LTP induction paradigms were used within 12 min after establishing the whole-cell configuration to prevent wash out effect on LTP induction [3]. The first protocol was pairing 80 presynaptic pulses at 2 Hz with postsynaptic depolarization at + 30 mV (referred to as the pairing protocol, [24]. The second protocol was paired 3 presynaptic stimuli which caused 3 excitatory postsynaptic potentials (EPSPs) (10 ms ahead) with 3 postsynaptic APs elicited by 0.5 nA, 10 ms current steps at 30 Hz, paired 15 times every 5s in the current clamp mode [32]. The third protocol was theta-burst stimulation (5 trains of burst with 4 pulses at 100 Hz, 200 ms interval; repeat 4 times with interval of 10 s) [3]. NMDA receptor-mediated component of EPSCs was pharmacologically isolated in ACSF containing: CNQX (20 μM), glycine (1 μM) and picrotoxin (100 μM). The patch electrodes contained (in mM) 102 cesium gluconate, 5 TEA chloride, 3.7 NaCl, 11 BAPTA, 0.2 EGTA, 20 HEPES, 2 MgATP, 0.3 NaGTP, and 5 QX-314 chloride (adjusted to pH 7.2 with CsOH). Neurons were voltage clamped at -30 mV and NMDA receptor-mediated EPSCs were evoked at 0.05 Hz. Access resistance was 15–30 MΩ and was monitored throughout the experiment.

### Pharmacological inhibitors

All chemicals and drugs including PD98059 and U0126 were obtained from Sigma (St. Louis, MO), except for QX-314, SP600125 and SB203580 that were from Tocris Cookson (Ellisville, MO). PD98059, U0126, SP600125 and SB203580 were dissolved in DMSO and diluted more than 1000-fold to give a final concentration in intracellular solution or ACSF. The diluted DMSO in intracellular solution or ACSF had no effect on synaptic transmission and plasticity.

### Data analysis and statistics

Data were collected and analyzed using pClamp 9.2 software (Axon Instruments). Data were discarded if access resistance changed more than 15% during an experiment. Rise times were determined between 10 and 90% of the peak amplitude of the evoked EPSC (eEPSCs). Decay times were measured from the peak to 37% of peak amplitude of eEPSCs. Statistical comparisons were performed using the Student's t-test. The level of significance was set at *P *< 0.05.
